# Waist Circumference Is Not Associated with Impaired Fasting Blood Glucose in a Sample of Mexican Children and Teenagers: Results from a State Screening Program

**DOI:** 10.3390/children8030172

**Published:** 2021-02-24

**Authors:** Edtna Jáuregui-Ulloa, Alejandro Gaytán-González, Mayra Elizalde-Villarreal, Esmeralda González-Navarro, Alberto Ocampo-Chavarría, Juan López-Taylor

**Affiliations:** 1 Institute of Applied Sciences for Physical Activity and Sport, Department of Human Movement Sciences, Education, Sport, Recreation and Dance, University Health Sciences Center, University of Guadalajara, Guadalajara 44430, Mexico; edtna.jauregui@academicos.udg.mx (E.J.-U.); alejandro.gaytan@cucs.udg.mx (A.G.-G.); 2 Cardiometabolic Program, Department of Preventive Medicine, Board for Health Prevention and Promotion, Jalisco Health Services, Guadalajara 44280, Mexico; ln.mayra.elizalde@gmail.com (M.E.-V.); gruposdeayudamutua@gmail.com (E.G.-N.); alberto.ocampo.ssj@gmail.com.mx (A.O.-C.); 3 Department of Human Reproduction, Infantile Growth and Development, University Health Sciences Center, University of Guadalajara, Guadalajara 44280, Mexico

**Keywords:** blood glucose, children, teenagers, waist circumference

## Abstract

A high waist circumference (WC) is used as a risk factor for impaired fasting blood glucose (IFG) in adults. This association is less studied in children and teenagers. The purpose of this study was to analyze the association between having a high WC and IFG by sex and age in a sample of Mexican children and teenagers. We analyzed the data of 12979 participants aged 5 to 17 years to calculate percentile references for uncorrected WC, corrected for height (WC/HT) and by height squared (WC/HT2) with quantile regression. A subsample of 2309 participants with fasting blood glucose samples (FBG), WC, WC/HT and WC/HT2 values was analyzed with logistic regression. A high WC, WC/HT, and WC/HT2 were considered at the sex- and age-specific 90th percentile from the subsample. The IFG was considered as FBG ≥100 mg/dL. Having a high WC, WC/HT, nor WC/HT2 was not significantly associated with IFG for either sex and age group (all *p* > 0.05). A high WC, either on its uncorrected or corrected for height values, was not an important assessment for predicting IFG in a sample of Mexican children and teenagers. This study provides percentile reference values specific for sex and age.

## 1. Introduction

The prevalence of obesity and its associated comorbidities, such as type 2 diabetes (T2D), and cardiovascular diseases, has increased worldwide [1,2,3]. This pattern was observed in children and teenagers as well [3,4,5,6]. In Mexico, the national prevalence of obesity increased from 14.6% to 17.5% in children aged 5 to 11 years and from 13.3% to 14.6% in teenagers aged 12 to 19 years from 2012 to 2018 [7]. The statistics of comorbidities associated with obesity, such as T2D, are scarce in Mexican children [8]. However, due to T2D has become the leading health problem in Mexico and the early onset of T2D has increased [8], it raises the concern about screening and prevention strategies in childhood [2,4,9]. 

Screening is an essential component of preventing T2D [10]. The assessment of fasting blood glucose (FBG) concentrations is a commonly used tool, from which we can determine whether the patient could be diagnosed with T2D, with an impaired fasting blood glucose concentration (IFG), or with a normal concentration [11]. Early detection of IFG might allow determining the appropriate medical or lifestyle interventions [12]. However, in clinical practice and epidemiological studies, FBG tests are not always available. Therefore, other tools, such as anthropometric measurements, are used for screening purposes because they are more accessible and less invasive than blood testing [13,14,15,16]. Body mass index (BMI) is likely the most commonly used anthropometric indicator for health screening due to its association with developing some non-communicable diseases and health conditions, both in children and adults [3,17]. However, BMI might not be sensible to fat distribution, and studies suggest that stored fat location, specifically visceral fat, is also important for health outcomes [18,19].

On the other hand, the assessment of waist circumference is one of the most commonly reported anthropometric measurements to assess visceral or central fat accumulation [20,21]. Along the same lines, waist circumference is associated with metabolic syndrome and cardiovascular diseases [13,14,15,22,23,24,25,26,27,28], highlighting the relevance of this anthropometric indicator. Some authors suggest that the sex- and age-specific 90th percentile of waist circumference could be considered a cut point for determining central obesity in children and teenagers [29,30,31]. This cut point has been validated in some studies where children having a waist circumference at or above the 90th percentile were at higher risk for dyslipidemia, insulin resistance, and other metabolic alterations [24,25,27,31]. Nonetheless, the utility of waist circumference as a risk factor for IFG is less studied in children and teenagers [24,32,33].

The provision of reference values of waist circumference from large samples might help screen children and teenagers in a clinical setting [31,34]. Previous studies reported percentile references in Mexican-American [35,36] and Mexican samples [37]. However, some did not report the waist circumference corrected for height or height squared, and they only reported 10th, 25th, 50th, 75th, and 90th percentiles. The correction for height might account for differences in height and changes during growth, whereas deciles and quartiles would help categorize and track patients better. Therefore, this study had three objectives: (1) Describe the waist circumference values by sex and age, (2) Develop percentile reference values for waist circumference by sex and age, and (3) Analyze the association between having a high waist circumference and IFG by sex and age in a sample of Mexican children and teenagers. Regarding this last objective, we hypothesized that a high waist circumference would be associated with a higher risk of having IFG.

## 2. Materials and Methods

### 2.1. Participants

This study came from the state program “*Módulos de Bienestar Familiar*” for health screening in Jalisco, Mexico, in 2018. The screening consisted of gathering information about health-related risk factors (e.g., anthropometric measurements, physical activity, nutritional habits) and blood samples for FBG assessment. All participants were deemed healthy with no previous medical diagnosis for any disease and without physical limitations by a medical questionnaire. 

**Figure 1:**
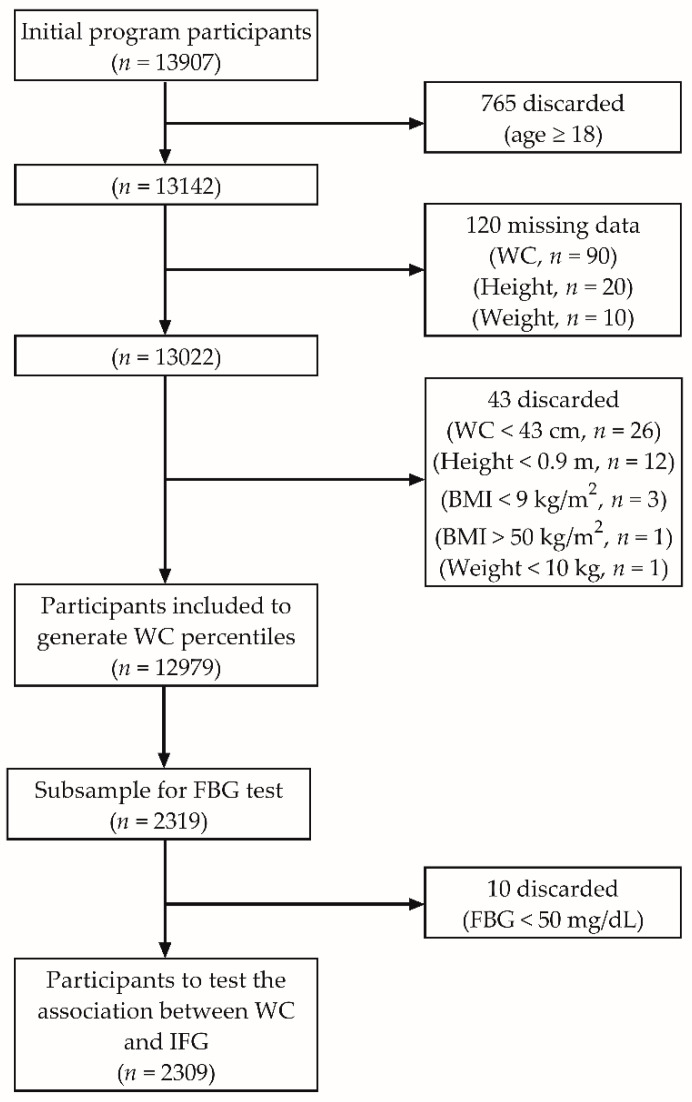
Participants flowchart. BMI: Body mass index; FBG: Fasting blood glucose; IFG: Impaired fasting blood glucose; WC: Waist circumference.

The flowchart of included participants is shown in Figure 1. We evaluated 13,907 participants aged 5 to 34 years from a sequential sample in schools or health centers/clinics throughout the state. However, we analyzed the data of participants aged <18 years (*n* = 13,142). All children and teenagers gave their assent to participate, and their parents/guardians gave their written informed consent before any assessment was carried out. After discarding participants’ records with missing data for waist circumference, body weight, or height, and those with unlikely values for waist circumference (<43 cm) [38], height (<0.9 m), body weight (<10 kg), or BMI (<9 or >50 kg/m^2^), it led to a sample size of 12,979 participants (6998 girls [53.9%], 5981 boys [46.1%]), from which we developed the percentile reference values. 

Blood samples were drawn when both the participant and his/her parent/guardian agreed to. Initially, 2351 out of 13142 participants agreed to provide a blood sample. After the filtering process mentioned above, 2319 participants remained with complete data. However, we discarded the records from participants having a blood glucose <50 mg/dL because this value appeared unlikely due to no participant reported hypoglycemia-related symptoms [39]. The final sample for this analysis was 2309, composed of 1271 girls (55%) and 1038 boys (45%) aged 5 to 17.9 year (Figure 1).

Participants were divided into single year categories (i.e., 5, 6, 7, 8, 9, 10, 11, 12, 13, 14, 15, 16, and 17) to calculate the percentile references and make comparisons by age within sex. On the other hand, the subsample participants were allocated into one of six age groups: 5 to 6.9, 7 to 8.9, 9 to 10.9, 11 to 12.9, 13 to 14.9, and 15 to 17.9 years. We chose this option because waist circumference did not differ significantly between immediate age groups (e.g., 5 vs. 6 year groups, 7 vs. 8 year groups), and the group combination would increase the sample size for each group and increase the statistical power for the logistic regression. 

### 2.2. Anthropometric Assessment

Health professionals took all the measurements following standardized protocols. Body weight was measured with a portable digital scale with a 0.1 kg precision (Omron HBF-514 C, OMRON Corporation, Kyoto, Japan), and height was measured in a standing position with a 1 cm precision (SECA 213, SECA, Hamburg, Germany). We calculated BMI and its corresponding z-score according to the BMI for age references published by the World Health Organization (WHO). Similarly, BMI was categorized according to the z-score cut points for underweight (<−2 SD), normal (>−2 and <1 SD), overweight (>1 SD), and obesity (>2 SD) [40,41].

Waist circumference was measured in a standing position at the end of exhalation with a rigid metal tape (Lufkin W606PM, Lufkin, NC, USA) positioned at the level of the midpoint between the iliac crest border and the lower costal border according to the WHO protocol [42], and recorded to the nearest centimeter. Waist circumference was reported as its uncorrected value in centimeters (WC), corrected for height (cm/m) (WC/HT), and corrected for height squared (cm/m^2^) (WC/HT2). All measurements were taken in the fasted state, with the child dressing in light clothing and without shoes.

### 2.3. Blood Glucose Assessment

FBG was determined from a capillary blood sample (finger) using disposable test strips and a glucometer (Accu-Check Active^®^, Roche DC Mexico S.A. de C.V.). The participants and their parents or guardians were instructed to let twelve hours lapse without food intake before the assessment. Impaired fasting blood glucose (IFG) was considered any concentration ≥100 mg/dL [11].

### 2.4. Statistical Analysis

Continuous data were analyzed for normal distribution with the Shapiro-Wilk test, suggesting all variables had a no-normal distribution (all *p* < 0.05). Therefore, data were reported as median (25th–75th percentile). IFG was reported as frequency counts (n) and percentage (%). Continuous variables were compared by age within sex with the Kruskal-Wallis H test. For the subsample, Dunn’s test was performed as post hoc after the Kruskal-Wallis H test. IFG was compared by sex and among age groups with X^2^ test of independence and t-test for proportions with Bonferroni adjustment as post hoc. The *p*-value for sex comparisons was calculated with Fisher’s exact test. Eta squared (η^2^) and phi statistic (φ) were calculated to determine the effect size of comparisons with Kruskal-Wallis H and X^2^ tests, respectively. Both η^2^ and φ are dimensionless statistics and range from 0 to 1. A small, medium and large effect size were considered if η^2^ was 0.01, 0.06, and 0.14, respectively; for φ the cut points were 0.1, 0.3, and 0.5, respectively [43]. Any effect size below the small effect size cut point was considered trivial [44]. We used quantile regression to calculate 5th, 10th, 15th, 20th, 25th, 30th, 40th, 50th, 60th, 70th, 75th, 80th, 85th, 90th, 95th percentiles from WC, WC/HT, and WC/HT2 specific by sex and age.

To determine the association of age and WC, WC/HT, and WC/HT2, we performed linear and segmented linear regressions, and we reported the one with the best fit. We set no constraint to the segmented linear regression to determine the slopes for the two regressed sections. 

Finally, we analyzed if having a high waist circumference was associated with IFG in the subsample of 2309 children and teenagers. A high waist circumference was considered at or above the 90th sex- by age group-specific percentile coming from the subsample [27,29,37]. We performed binomial logistic regressions, setting high waist circumference as the predictor variable and IFG as the outcome variable. This analysis had one unadjusted model (model 1) and one adjusted for BMI categories (model 2). Results from binomial logistic regressions are reported as odds ratios (OR) and 95% confidence intervals (95% CI). The comparisons and results from logistic regression were deemed significant at a *p*-value ≤ 0.05. Data were analyzed with SPSS^®^v.26 (IBM Corp., Armonk, NY, USA) for Windows^®^. Linear regressions, segmented linear regressions and graphs were performed with GraphPad^®^ Prism v7.05 (GraphPad Software Inc., La Jolla, CA, USA) for Windows^®^.

## 3. Results

### 3.1. Anthropometric Comparisons by Age 

**Figure 2:**
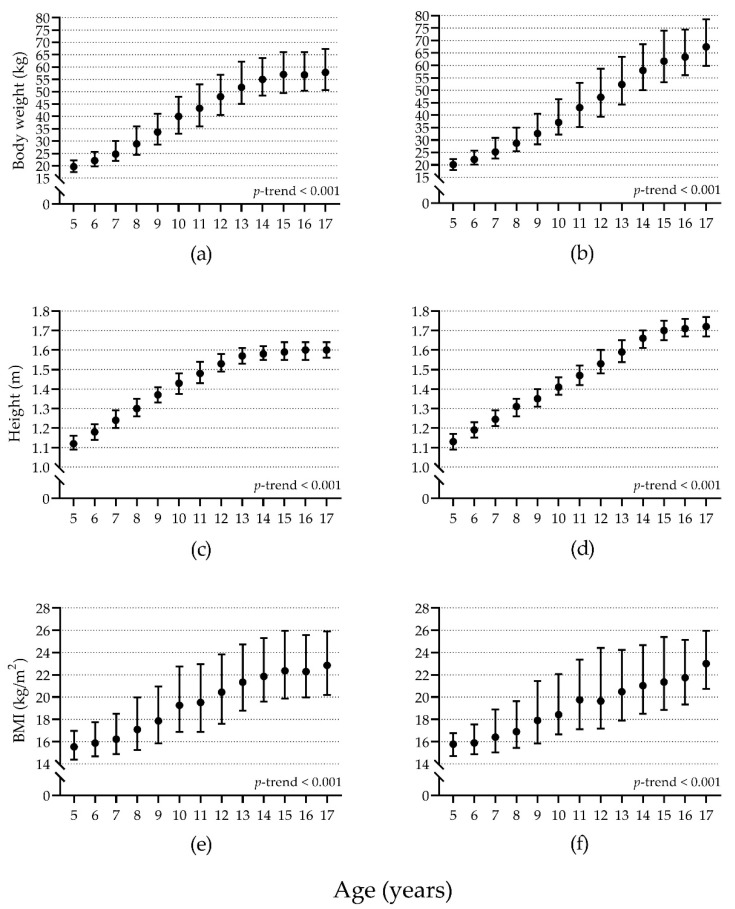
Body weight (**a**,**b**), height (**c**,**d**), and BMI (**e**,**f**) comparison by age in girls (**a**,**c**,**e**) (*n* = 6998) and boys (**b**,**d**,**f**) (*n* = 5981). Solid circles represent the median values; whiskers represent the 25th and 75th percentiles. *p*-trend calculated with the Kruskal-Wallis H test. BMI: Body mass index.

As expected, body weight, height, and BMI were significantly higher as age increased, for both girls and boys (all *p* < 0.001) (Figure 2); showing a large effect size for age (Girls: body weight, η^2^ = 0.610; height, η^2^ = 0.731; BMI, η^2^ = 0.291; Boys: body weight, η^2^ = 0.670; height, η^2^ = 0.842; BMI, η^2^ = 0.249). BMI categories differed significantly between girls and boys. Girls showed a significantly lower prevalence of underweight and obesity than boys, but a significantly higher prevalence of normal BMI and overweight. All comparisons showed trivial effect sizes (Table 1).

**Figure 3:**
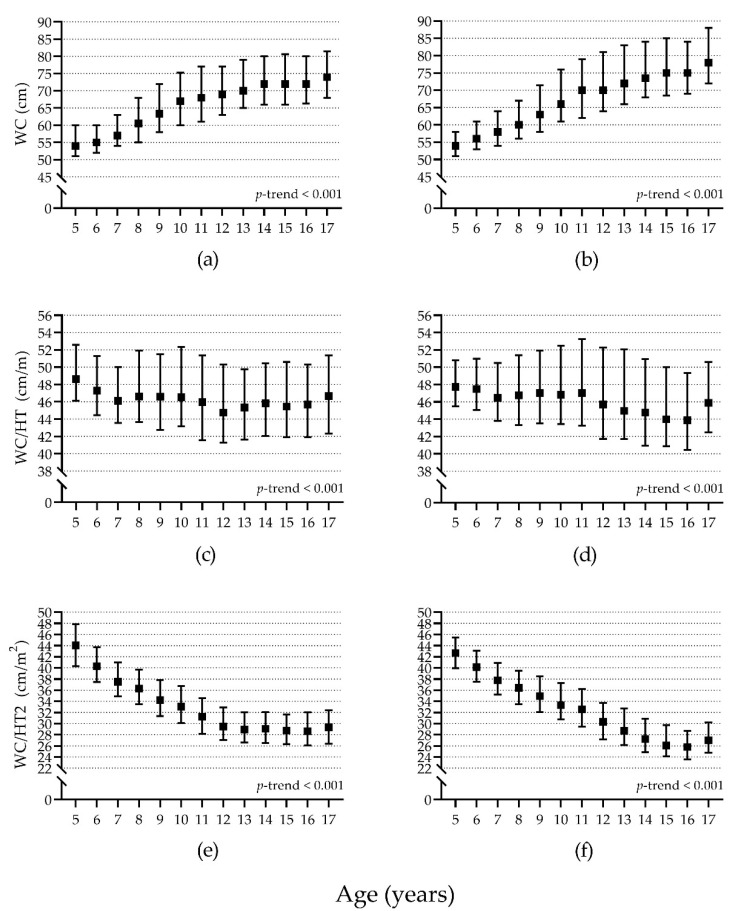
Waist circumference (WC) (**a**,**b**), waist circumference corrected for height (WC/HT) (**c**,**d**), and waist circumference corrected for height squared (WC/HT2) (**e**,**f**) comparison by age in girls (**a**,**c**,**e**) (*n* = 6998) and boys (**b**,**d**,**f**) (*n* = 5981). Solid squares represent the median values; whiskers represent the 25th and 75th percentiles. *p*-trend calculated with the Kruskal-Wallis H test.

Waist circumference (cm) was significantly larger as age increased and showed a large effect size for both girls (η^2^ = 0.299) and boys (η^2^ = 0.361) (Figure 3a,b). The opposite pattern was observed for both WC/HT and WC/HT2. These values were significantly smaller as age increased for both girls and boys. However, this pattern was less marked for WC/HT, with a small to moderate effect size (girls, η^2^ = 0.017; boys, η^2^ = 0.031) (Figure 3c,d); whereas it was more marked for WC/HT2, with a large effect size (girls, η^2^ = 0.404; boys, η^2^ = 0.498) (Figure 3e,f).

The segmented linear regression showed better results than linear regression for WC in girls and boys and WC/HT2 in girls. For the other analysis, linear regression estimated this association better. For girls, WC increased 2.6 cm per year until the 11.5 years, and 0.8 cm per year thereafter (R^2^ = 0.255); for boys, WC increased 2.8 cm per year until the 11.4 years, and 1.5 cm per year thereafter (R^2^ = 0.300). For WC/HT there was a decrease of 0.14 cm/m per year in girls (R^2^ = 0.006) and a decrease of 0.21 cm/m per year in boys (R^2^ = 0.011). For WC/HT2, there was a decrease of 1.74 cm/m^2^ per year until the 12.5 years, and a decrease of 0.04 cm/m^2^ per year thereafter in girls (R^2^ = 0.419); for boys, there was a decrease of 1.34 cm/m^2^ per year (R^2^ = 0.460) (Figure S1).

### 3.2. Waist Circumference Percentiles

Reference percentiles divided by sex and age can be found in Tables S1 and S2 for WC, Tables S3 and S4 for WC/HT, and Tables S5 and S6 for WC/HT2. 

### 3.3. Subsample Analysis and Fasting Blood Glucose

The age comparison within sex showed a similar pattern in the subsample as the one observed in the whole sample for body weight, height, BMI, WC, WC/HT, and WC/HT2 (Tables S7 and S8). Some BMI categories differed significantly among age groups. Normal and overweight categories differed in girls, and overweight and obesity categories differed in boys; all significant differences showed a small to moderate effect size (Table S9). FBG was significantly different among age groups and showed a small to moderate effect size for both girls (*p* < 0.001, η^2^ = 0.026) and boys (*p* = 0.001, η^2^ = 0.015). Median FBG ranged from 90 to 95 mg/dL in girls and 92 to 96 mg/dL in boys. For both sexes, the lowest median value was observed in the 15 to 17.9 years group. Whereas the highest median value was observed in the 9 to 10.9 and 11 to 12.9 years groups in girls, and in the 7 to 8.9 and 11 to 12.9 years groups in boys (Table 2). 

IFG was observed in 565 participants (24.5% [565/2309]). Girls showed a significantly lower percentage of IFG (22.0% [279/1271]) than boys (27.6% [286/1038]) (*p* = 0.002) but with a trivial effect size (φ = 0.065). Girls showed the highest percentage of IFG at the 11 to 12.9 years group (30.1%) and the lowest at the 15 to 17.9 years group (14.0%), with significant differences and a small to moderate effect size (φ = 0.146). For boys, the highest percentage was observed at the 11 to 12.9 years group (36.3%) and the lowest at the 15 to 17.9 years group (22.0%), with significant differences and a small to moderate effect size (φ = 0.117) (Table 2).

### 3.4. Waist Circumference and Impaired Fasting Blood Glucose Concentrations

Having a high WC was significantly associated with a higher risk of presenting IFG in girls in the 7 to 8.9 years group. Similarly, having a high WC/HT was significantly associated with IFG in girls in 7 to 8.9 and 13 to 14.9 years groups. Finally, having a high WC/HT2 was significantly associated with IFG in girls in 15 to 17.9 years group. Nonetheless, there were no significant associations after adjusting for BMI categories at any age group in girls (Table 3). For boys, having a high WC, WC/HT, or WC/HT2 was not significantly associated with IFG at any age group for either model (Table 4).

## 4. Discussion

The current study suggests that having a high waist circumference, either for uncorrected or corrected values, is not associated with IFG in a sample of Mexican children and teenagers. This result is contrary to what was previously reported in Mexican adults [45] and children [24]. One of the main differences between these two studies and the present one is the method to determine the cut points. Berber et al. [45] and López-Gonzalez et al. [24] used the receiver operating characteristic (ROC) curve method. In contrast, we followed a percentile-derived cut point according to the 90th percentile recommended for waist circumference in previous studies [27,29,37]. Similarly, the FBG results may differ between capillary and venous blood samples [46], explaining the different findings among these studies. 

Another possible explanation is that 79.2% of participants in the study of López-Gonzalez et al. were categorized as having overweight or obesity. In contrast, in our study, the combined prevalence of overweight and obesity was 39.2% (Table 1). Considering this is almost two-fold of our prevalence, it would be more likely to find an association due to the role of overweight and obesity over IFG [47]. 

Nonetheless, the study of Morandi et al. [33] came up with the same conclusions as we did in the present study. They did not find a significant association between WC or waist-to-height ratio (in this case, the same as WC/HT) and IFG in obese Italian children aged 8 to 18. The anatomic site where the waist circumference was assessed could be considered a factor that explains the differences among these studies. However, Harrington et al. [48] suggested that the measurement site does not affect the association between waist circumference and cardiometabolic parameters, including FBG, even though these different anatomical sites provided different absolute values for waist circumference. Therefore, it appears unlikely to explain the differences between the analyzed studies.

Monitoring FBG in children is clinically relevant considering that about 10% of children and teenagers would have IFG [24,49], and IFG might migrate to prediabetes and T2D in early ages, an emerging problem in Mexico [7,8]. Taking anthropometric measurements, like waist circumference, may help monitor risk factors for IFG and are more available and cheaper than blood samples [13,14,15,16]. However, our results do not support this idea in Mexican children and teenagers (Table 3 and Table 4). Nonetheless, other variables like hormonal changes observed at puberty may explain this increase in FBG and not necessarily related to waist circumference [50]. For instance, puberty may predispose a decline in insulin sensitivity between 25 to 30% from the prepuberal to the puberal stage, leading to a compensatory increase in insulin secretion compared with adults [51,52]. These changes may lead to insulin resistance in the youth, with a prevalence between 3.1 and 44%, depending on the definition and the population [53]. Therefore, it is essential to follow up on these hormonal changes during maturation to discard any pathological changes that led to metabolic disorders.

The lack of association between waist circumference and IFG in this study might be attributable to other risk factors such as physical activity levels and nutritional status. Evidence suggests that high levels of moderate-to-vigorous physical activity are significantly associated with lower values of waist circumference, systolic blood pressure, fasting insulin and triglycerides [54]. Moreover, other studies highlight the importance of considering sedentary behavior as a risk factor. For instance, a shorter time expended in sedentary activities is associated with a smaller waist circumference and lower adiposity in children [55,56]. Regarding the nutritional status, it is known that a high BMI (overweight or obesity) is associated with metabolic disorders such as hyperglycemia and hypertriglyceridemia in children [3,17]. Because of this association, we adjusted the logistic regressions for BMI categories. 

Our data suggest an age-related change for WC, WC/HT, and WC/HT2 for both girls and boys (Figure 3 and Figure S1). However, the association between age and WC/HT is weaker than in the other two parameters. We consider that this result might support the idea that the waist-to-height ratio cut point of 0.5 m/m (in this case is the same as WC/HT, 50 cm/m) previously recommended as a “global indicator” for all ages, sexes, and ethnicities [57] might indeed apply to girls and boys due to it is less variable in comparison to WC and WC/HT2 in different ages. However, a post hoc analysis using this cut point revealed, again, no significant association in this sample (data not shown). Additionally, we observed that this cut point was usually between the 70th and 75th percentiles in our sample (Tables S3 and S4), indicating that maybe this cut point was not high enough to detect a high waist circumference or that our sample had a large enough waist circumference to impede this cut point to detect cases.

Regarding the percentile references, we found that the WC percentiles had larger values than the previously reported in the Mexican population ten years ago by Klunder et al. [37]. Still, the estimated WC/HT values were similar. This increase in WC might be explained by the rise in overweight and obesity prevalence in Mexican children in the last years. For instance, the combined prevalence of overweight and obesity in children aged 5 to 11 years increased from 34.4% in 2012 to 35.5% in 2018, and from 34.9% in 2012 to 38.4% in 2018 for teenagers aged 12 to 19 years [7]. 

We can find several percentile references in the literature having different statistical approaches and sample sizes. Klunder et al. [37] used percentile regression in a sample 3376 of Mexican children and teenagers aged 6 to 16 years. Fernandez et al. [36] and Messiah et al. [35] used percentile regression in a nationwide representative sample of children aged 2 to 18 years in the United States (Mexican-American children included). Marrodan Serrano et al. [34] used the Lambda Mu Sigma (LMS) method to calculate percentile references using data of 13289 participants aged 6 to 18 years from five Latin-American countries (Mexico included). Xi et al. [31] analyzed data of ≈113,000 participants aged 4 to 20 years from eight countries (Mexico not included) using the Generalized Additive Model for Location, Scale, and Shape (GAMLSS). This type of study is easy to find in the literature; however, most of them reported the uncorrected waist circumference and, to our knowledge, only one reported reference values in Mexican children [37]. In this regard, the present study offers percentile references for uncorrected and corrected waist circumference in a large sample of Mexican children and teenagers.

Our findings should be interpreted considering their limitations. Firstly, the cross-sectional design does not allow us to infer causality; therefore, longitudinal studies may help determine waist circumference performance to predict the development of IFG in children and teenagers. Secondly, we did not evaluate the maturation stage; the lack of this variable may explain the observed differences among studies as maturation may occur at different ages and affect body composition, anthropometric measurements and blood markers [50,58,59]. Similarly, and as mentioned previously, physical activity may affect the insulin and blood glucose levels, independently of the adiposity, including waist circumference as an indirect measurement [60]. Therefore, the lack of this variable in this study may limit its association. Again, longitudinal studies should investigate the interaction between anthropometric measurements, maturation state, physical activity, and FBG. 

Thirdly, the blood sample was taken from finger capillaries, and such values may differ from those observed by venous blood samples [46], possibly leading to different results. Additionally, the assessment of FBG by reactive strips and glucometers might be inaccurate. In this regard, the ISO 15197: 2013 requires that 95% of the results of FBG by these devices must be ±15 mg/dL of the reference value if it is <100 mg/dL, or within 15% of the reference value if it is ≥100 mg/dL [61]. If we assume a participant has a reference value of 110 mg/dL, the obtained measurement might range from a normal 94 to a diabetic 126 mg/dL. Nonetheless, the method we used has reported that a deviation of 8.4% from the reference value includes at least 95% of the assessments [62]. Suggesting that the range of accuracy is narrower than the required by the ISO 15197: 2013. For example, the same 110 mg/dL might range from 101 to 119 mg/dL. Still, the participants with a FBG between 91 and 109 mg/dL might be wrongly classified as either a normal or IFG due to the measurement error. 

Fourthly, the analysis by age groups instead of age alone may affect the results; however, this combination was necessary to increase the sample size and increase the logistic regression’s statistical power. Whereas the subsample was large enough, the analysis of each sex- by age-specific group consisted of taking the highest tenth part of that group (≥90th percentile) and looking for IFG. Such analysis was impossible to do without forming age groups. Another important limitation is that the subsample with FBG assessments was not randomly selected; instead, the inclusion consisted of participants’ willingness and guardians’ approval to undergo this assessment. This subsample represents 17.9% of participants aged <18 y in this study. Therefore, these results’ generalizability might be limited for its low representativeness, even though the sample size is large.

Finally, some of the strengths of this study are that we had a large sample size and the analysis was sex- and age-specific for three ways of reporting waist circumference, including the unadjusted and adjusted for BMI categories, allowing us to control for the possible influence of BMI over IFG (Table 3 and Table 4). 

## 5. Conclusions

In this study, having a high waist circumference, either on its uncorrected or corrected for height values, was not an important assessment for predicting IFG in a sample of Mexican children and teenagers during a screening program. This study provides percentile reference values for uncorrected and corrected waist circumference specific for sex and age from a large sample of Mexican children and teenagers aged 5 to 17.

## Figures and Tables

**Table 1:** Comparison of body mass index categories between girls and boys.

	Total (*n* = 12979)		Girls (*n* = 6998)		Boys (*n* = 5981)		*p*-Value ^§^	φ
	*n*	%	*n*	%	*n*	%		
Underweight	320	2.5	150	2.1	170	2.8	0.012	0.022
Normal	7570	58.3	4192	59.9	3378	56.5	<0.001	0.035
Overweight	2796	21.5	1604	22.9	1192	19.9	<0.001	0.036
Obesity	2293	17.7	1052	15.0	1241	20.7	<0.001	0.075

^§^*p*-value for comparisons between girls and boys (X^2^ test of independence plus Fisher’s exact test).

**Table 2:** Comparison of fasting blood glucose (FBG) and impaired fasting blood glucose concentration (IFG) in the subsample of participants by sex and age group (*n* = 2309).

	Age Group (Years)						*p*-Trend ^§^
	5 to 6.9	7 to 8.9	9 to 10.9	11 to 12.9	13 to 14.9	15 to 17.9	
	Girls (*n* = 1271)						
*n*	100	174	200	216	232	349	
FBG (mg/dL) ^†^	92 ^a,b,c^ (85–97)	93 ^a,c^ (86–98)	95 ^a^ (89–102)	95 ^a,c^ (85–101)	92 ^b,c^ (84–98)	90 ^b^ (84–96)	<0.001
IFG ^‡^	18 ^a,b^ (18.0)	40 ^a,b^ (23.0)	57 ^b^ (28.5)	65 ^b^ (30.1)	50 ^a,b^ (21.6)	49 ^a^ (14.0)	<0.001
	**Boys (*n* = 1038)**						
***n***	**87**	**137**	**178**	**190**	**205**	**241**	
FBG (mg/dL)	94 ^a,b^ (82–100)	96 ^a^ (88–102)	95 ^a^ (90–102)	96 ^a^ (87–103)	94 ^a,b^ (86–99)	92 ^b^ (85–99)	0.001
IFG	24 ^a,b^ (27.6)	43 ^a,b^ (31.4)	50 ^a,b^ (28.1)	69 ^b^ (36.3)	47 ^a,b^ (22.9)	53 ^a^ (22.0)	0.014

^†^ Data expressed as median (25th–75th percentile). ^‡^ Considered as a glucose concentration ≥100 mg/dL. Data expressed as n (%). ^§^ Calculated with Kruskal-Wallis H test for continuous variables or with X^2^ test for categorical variables. Different letters within variables denote significant differences (*p* ≤ 0.05) among age groups (Dunn’s post hoc test for continuous variables; *t*-test for independent proportions with Bonferroni adjustment for categorical variables).

**Table 3:** Association between having a high (≥90th percentile) waist circumference (WC), waist circumference corrected for height (WC/HT), or waist circumference corrected for height squared (WC/HT2) and impaired fasting blood glucose (≥100 mg/dL) in girls by age group (*n* = 1271).

Age Group (Years)	Cut Point ^†^	Participants above the Cut Point (*n*)	Model 1		Model 2	
			OR (95% CI) ^‡^	*p*-Value	OR (95% CI)	*p*-Value
	WC (cm)					
5 to 6.9	66.3	10	2.14 (0.50–9.24)	0.31	1.87 (0.26–13.3)	0.53
7 to 8.9	72.9	18	3.10 (1.13–8.49)	0.03	1.74 (0.39–7.72)	0.47
9 to 10.9	81.0	21	2.05 (0.81–5.16)	0.13	1.42 (0.45–4.53)	0.55
11 to 12.9	87.0	22	0.86 (0.32–2.30)	0.76	0.52 (0.15–1.83)	0.31
13 to 14.9	89.0	25	1.48 (0.58–3.78)	0.41	1.25 (0.34–4.62)	0.74
15 to 17.9	90.0	35	1.62 (0.67–3.94)	0.29	0.75 (0.23–2.46)	0.64
	WC/HT (cm/m)					
5 to 6.9	55.71	10	2.14 (0.50–9.24)	0.31	1.84 (0.36–9.49)	0.47
7 to 8.9	54.69	18	3.10 (1.13–8.49)	0.03	1.59 (0.32–7.93)	0.57
9 to 10.9	57.66	20	1.78 (0.69–4.62)	0.24	1.15 (0.36–3.69)	0.82
11 to 12.9	56.13	22	0.86 (0.32–2.30)	0.76	0.56 (0.16–1.89)	0.35
13 to 14.9	55.15	24	2.44 (1.00–5.98)	0.05	3.14 (0.92–10.7)	0.07
15 to 17.9	56.55	35	1.62 (0.67–3.94)	0.29	0.79 (0.25–2.49)	0.68
	WC/HT2 (cm/m^2^)					
5 to 6.9	48.63	10	0.48 (0.06–4.02)	0.50	0.44 (0.05–3.81)	0.46
7 to 8.9	43.59	18	2.37 (0.85–6.60)	0.10	1.30 (0.37–4.60)	0.68
9 to 10.9	41.25	20	1.40 (0.53–3.71)	0.50	0.87 (0.28–2.70)	0.82
11 to 12.9	37.11	22	0.66 (0.23–1.86)	0.43	0.49 (0.15–1.56)	0.23
13 to 14.9	35.14	24	1.58 (0.62–4.05)	0.34	1.50 (0.51–4.46)	0.46
15 to 17.9	36.05	35	2.37 (1.04–5.42)	0.04	1.61 (0.58–4.47)	0.36

^†^ 90th percentile value was considered the cut point. Participants below the 90th percentile were the reference group. ^‡^ Odds ratios (95% confidence intervals). Model 1: Unadjusted logistic regression; Model 2: Logistic regression adjusted for BMI categories.

**Table 4:** Association between having a high (≥90th percentile) waist circumference (WC), waist circumference corrected for height (WC/HT), or waist circumference corrected for height squared (WC/HT2) and impaired fasting blood glucose (≥100 mg/dL) in boys by age group (*n* = 1038).

Age Group (Years)	Cut Point ^†^	Participants above the Cut Point (*n*)	Model 1		Model 2	
			OR (95% CI) ^‡^	*p*-Value	OR (95% CI)	*p*-Value
	WC (cm)					
5 to 6.9	68.0	10	1.14 (0.27–4.84)	0.86	1.29 (0.24–6.88)	0.77
7 to 8.9	75.0	14	0.57 (0.15–2.14)	0.40	0.75 (0.14–4.17)	0.74
9 to 10.9	87.0	18	1.32 (0.47–3.73)	0.60	1.25 (0.35–4.49)	0.73
11 to 12.9	91.2	19	1.31 (0.50–3.44)	0.58	1.78 (0.56–5.62)	0.33
13 to 14.9	90.0	21	1.40 (0.51–3.82)	0.52	0.79 (0.19–3.22)	0.74
15 to 17.9	98.0	29	0.71 (0.26–1.97)	0.51	0.40 (0.11–1.47)	0.17
	WC/HT (cm/m)					
5 to 6.9	57.63	9	0.73 (0.14–3.78)	0.71	0.80 (0.14–4.74)	0.81
7 to 8.9	56.77	14	0.86 (0.25–2.92)	0.81	1.45 (0.30–7.00)	0.64
9 to 10.9	60.52	18	1.32 (0.47–3.73)	0.60	1.25 (0.35–4.49)	0.73
11 to 12.9	60.56	19	1.03 (0.38–2.74)	0.96	1.28 (0.39–4.24)	0.68
13 to 14.9	55.93	21	1.40 (0.51–3.82)	0.52	0.92 (0.25–3.31)	0.89
15 to 17.9	57.54	25	0.65 (0.21–1.98)	0.45	0.35 (0.09–1.45)	0.15
	WC/HT2 (cm/m^2^)					
5 to 6.9	49.43	9	#		#	
7 to 8.9	43.83	14	0.86 (0.25–2.92)	0.81	1.35 (0.31–5.94)	0.69
9 to 10.9	42.44	18	0.98 (0.33–2.92)	0.98	0.81 (0.22–2.98)	0.75
11 to 12.9	39.15	19	1.03 (0.38–2.74)	0.96	1.28 (0.39–4.24)	0.68
13 to 14.9	35.84	21	1.40 (0.51–3.82)	0.52	1.02 (0.31–3.35)	0.98
15 to 17.9	33.87	25	0.45 (0.13–1.58)	0.21	0.26 (0.07–1.07)	0.06

^†^ 90th percentile value was considered the cut point. Participants below the 90th percentile were the reference group. ^‡^ Odds ratios (95% confidence intervals). # OR (95% CI) were not calculated due to cases were absent in one of the compared groups. Model 1: Unadjusted logistic regression; Model 2: Logistic regression adjusted for BMI categories.

## Data Availability

Restrictions apply to the availability of these data. Data was obtained from Jalisco Health Services and are available from Dirección de Generación de Recursos Profesionales, Investigación y Desarrollo; Servicios de Salud Jalisco. Data should be requested to this entity by writing an email to informespsaa@gmail.com.

## Supplementary Materials

The following are available online at https://www.mdpi.com/2227-9067/8/3/172/s1, Figure S1: Correlation between age and waist circumference (WC), waist circumference corrected for height (WC/HT), and waist circumference corrected for height squared (WC/HT2) in girls and boys; Table S1: Waist circumference (cm) percentiles in girls by age; Table S2: Waist circumference (cm) percentiles in boys by age; Table S3: Waist circumference corrected for height (cm/m) percentiles in girls by age; Table S4: Waist circumference corrected for height (cm/m) percentiles in boys by age; Table S5: Waist circumference corrected for height squared (cm/m^2^) percentiles in girls by age; Table S6: Waist circumference corrected for height squared (cm/m^2^) percentiles in boys by age; Table S7: Main characteristics by sex and age group for the subsample; Table S8: Waist circumference values by sex and age group for the subsample; Table S9: Body mass index categories by sex and age group for the subsample.
