# Supplementary material for: Waist Circumference Is Not Associated with Impaired Fasting Blood Glucose in a Sample of Mexican Children and Teenagers: Results from a State Screening Program
Source: Children (Basel). 2021 Feb 24;8(3):172. doi: 10.3390/children8030172 (PMC7996355; doi:10.3390/children8030172)
Supplement: Supplementary file 1 [file children-08-00172-s001.pdf]

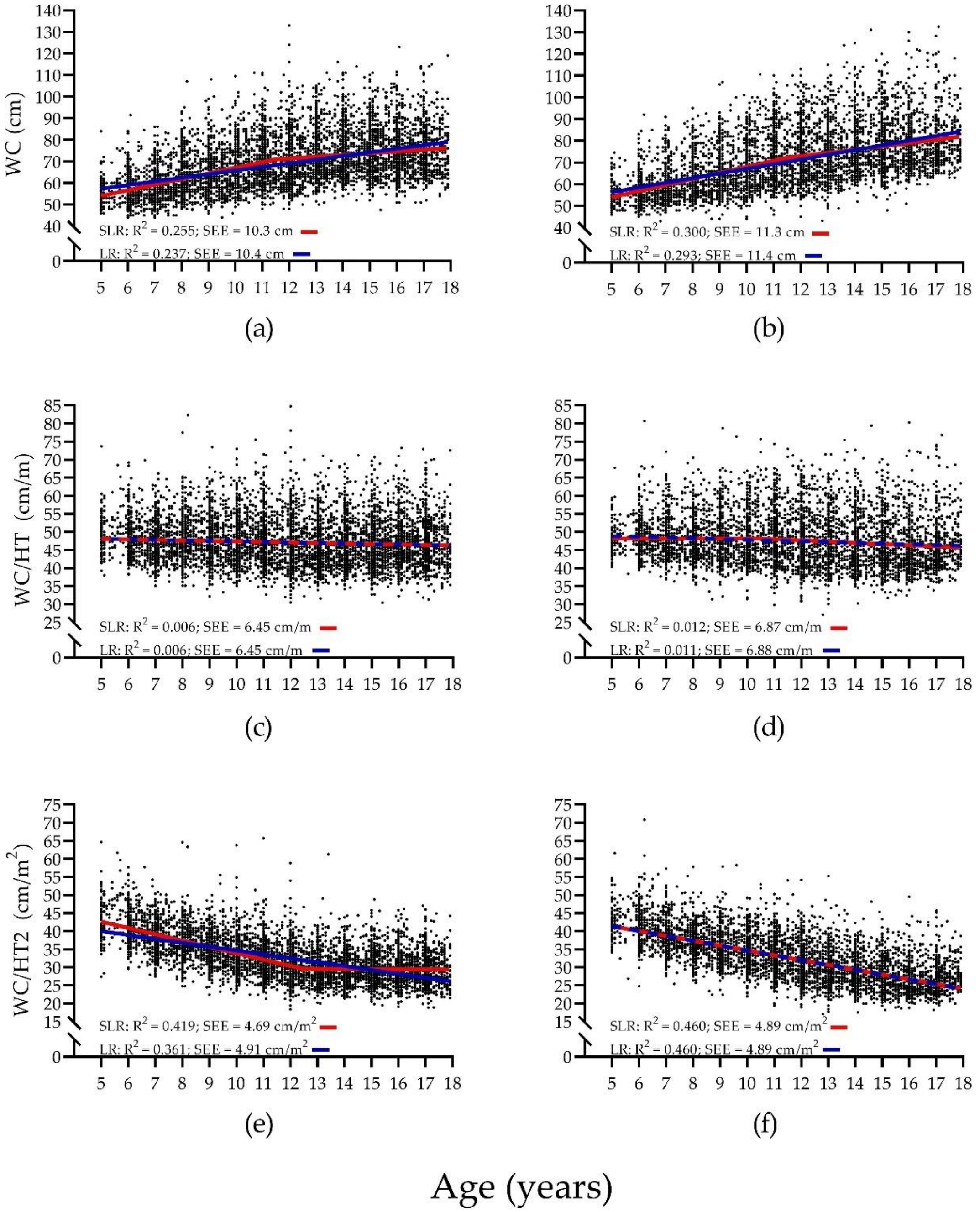

Figure S1. Correlation between age and waist circumference (WC) (a, b), waist circumference corrected for height (WC/HT) (c, d), and waist circumference corrected for height squared (WC/HT2) (e, f) in girls (a, c, e) (*n* = 6998) and boys (b, d, f) (*n* = 5981). LR: Linear regression (blue line); SEE: Standard error or estimation; SLR: Segmented linear regression (red line). The overlapping between SLR and LR is denoted by semicontinuous regression lines in graphs (c, d, f).

Table S1. Waist circumference (cm) percentiles in girls by age (n = 6998).

	Age (years)												
**Percentile**	**5** (n = 161)	**6** (n = 477)	**7** (n = 491)	**8** (n = 480)	**9** (n = 516)	**10** (n = 549)	**11** (n = 525)	**12** (n = 699)	**13** (n = 653)	**14** (n = 666)	**15** (n = 705)	**16** (n = 620)	**17** (n = 456)
5th	48.0	48.0	49.0	51.0	52.0	53.5	55.0	56.0	59.0	61.0	60.0	60.0	62.0
10th	49.0	49.8	51.0	52.5	54.0	56.0	57.0	58.1	61.0	62.5	62.5	62.0	63.0
15th	49.0	50.0	52.0	54.0	55.5	58.0	59.0	60.0	62.5	64.0	63.5	64.0	65.0
20th	50.5	51.0	53.0	55.0	57.0	59.0	60.0	61.0	64.0	65.0	65.5	65.0	66.0
25th	51.0	52.0	54.0	55.0	58.0	60.0	61.0	63.0	65.0	66.0	66.0	66.3	68.0
30th	51.8	53.0	55.0	56.5	59.0	62.0	62.0	64.0	66.0	67.5	67.0	68.0	69.0
40th	53.0	54.0	56.0	59.0	61.0	64.0	65.0	66.0	68.0	69.8	70.0	70.0	71.9
50th	54.0	55.0	57.0	60.5	63.5	67.0	68.0	69.0	70.0	72.0	72.0	72.0	74.0
60th	56.3	57.0	59.0	63.0	67.0	69.0	71.0	72.0	73.0	75.0	74.5	75.0	77.0
70th	59.0	60.0	61.0	67.0	70.0	73.0	74.5	75.0	77.0	78.0	78.8	78.0	80.0
75th	60.0	60.0	63.0	68.0	72.0	75.0	77.0	77.0	79.0	80.0	80.5	81.0	81.5
80th	60.0	62.0	64.0	70.0	74.0	78.0	79.0	80.0	81.0	82.0	83.0	83.0	83.0
85th	62.0	64.0	67.0	73.0	76.0	80.5	82.0	83.0	84.0	84.0	85.0	85.0	86.0
90th	64.0	67.0	70.0	77.0	79.0	84.0	85.0	87.0	88.0	88.0	89.0	89.0	89.5
95th	69.5	70.0	75.0	81.5	85.0	87.0	90.0	92.0	94.0	93.0	95.0	96.0	95.0

Table S2. Waist circumference (cm) percentiles in boys by age (n = 5981).

Percentile	Age (years)												
	**5 (n = 164)**	**6 (n = 442)**	**7 (n = 422)**	**8 (n = 463)**	**9 (n = 495)**	**10 (n = 502)**	**11 (n = 554)**	**12 (n = 628)**	**13 (n = 530)**	**14 (n = 521)**	**15 (n = 536)**	**16 (n = 408)**	**17 (n = 316)**
5th	48.0	48.0	50.0	51.5	53.0	55.0	56.0	57.5	59.0	62.0	62.0	64.0	66.5
10th	49.0	50.0	52.0	53.0	54.0	57.0	58.0	60.0	62.0	64.0	64.0	66.0	69.5
15th	50.0	51.2	52.0	54.5	56.0	59.0	60.0	61.5	63.0	65.0	66.4	67.0	70.5
20th	50.0	52.5	53.0	55.0	57.0	60.0	61.0	63.0	64.0	66.0	68.0	68.0	71.0
25th	51.0	53.0	54.0	56.0	58.0	61.0	62.0	64.0	66.0	68.0	68.5	69.0	72.0
30th	51.0	53.5	54.0	57.0	59.0	62.0	63.0	64.7	67.0	69.0	69.5	70.0	74.0
40th	52.0	55.0	56.0	58.0	61.0	64.0	66.0	67.0	69.0	71.0	72.0	72.0	76.0
50th	54.0	56.0	58.0	60.0	63.0	66.0	70.0	70.0	72.0	73.5	75.0	75.0	78.0
60th	55.0	58.0	60.0	62.0	66.0	69.0	73.0	74.0	75.0	77.0	78.5	78.0	80.5
70th	57.0	60.0	62.0	65.5	69.0	73.0	77.0	78.0	80.0	81.5	83.0	81.5	86.0
75th	58.0	61.0	64.0	67.0	71.4	76.0	79.0	81.0	83.0	84.0	85.0	84.0	88.0
80th	59.0	63.0	66.0	69.0	75.0	79.0	82.5	84.0	85.5	87.0	87.5	87.0	90.0
85th	60.5	64.5	68.5	72.5	78.0	82.0	86.0	87.0	89.0	91.0	90.5	91.0	95.0
90th	64.0	67.0	73.0	76.0	82.0	86.0	90.0	92.0	92.0	95.0	97.0	95.0	100.0
95th	68.0	73.0	79.0	81.0	87.0	90.1	95.0	97.0	99.0	101.0	103.0	102.0	107.0

Table S3. Waist circumference corrected for height (cm/m) percentiles in girls by age (*n* = 6998).

	Age (years)												
**Percentile**	**5 (*n* = 161)**	**6 (*n* = 477)**	**7 (*n* = 491)**	**8 (*n* = 480)**	**9 (*n* = 516)**	**10 (*n* = 549)**	**11 (*n* = 525)**	**12 (*n* = 699)**	**13 (*n* = 653)**	**14 (*n* = 666)**	**15 (*n* = 705)**	**16 (*n* = 620)**	**17 (*n* = 456)**
5th	41.74	41.07	40.16	39.68	38.81	39.10	38.30	37.20	37.86	38.46	37.97	37.91	37.80
10th	43.36	42.11	41.41	41.06	40.58	40.13	39.31	38.93	39.35	39.74	39.22	39.16	39.51
15th	44.25	43.31	42.31	42.06	41.20	41.04	40.14	40.00	40.13	40.60	40.25	40.24	40.51
20th	45.37	43.86	43.08	42.75	41.98	42.55	40.94	40.63	40.79	41.23	41.10	41.07	41.40
25th	46.15	44.49	43.55	43.66	42.75	43.17	41.56	41.28	41.67	42.04	41.88	41.91	42.32
30th	46.43	44.91	44.00	44.44	43.48	43.91	42.28	41.94	42.38	42.73	42.56	42.63	43.21
40th	47.50	46.09	45.08	45.49	44.85	45.10	43.84	43.42	43.69	44.16	43.90	44.03	44.97
50th	48.62	47.29	46.12	46.62	46.60	46.54	45.95	44.74	45.34	45.80	45.45	45.68	46.64
60th	50.00	48.39	47.44	48.51	48.06	48.57	47.71	46.45	47.06	47.20	47.10	47.13	48.39
70th	51.72	50.00	49.15	50.39	50.34	51.01	50.00	49.01	49.03	49.36	49.07	49.07	50.63
75th	52.54	51.28	50.00	51.89	51.48	52.33	51.35	50.32	49.70	50.45	50.63	50.31	51.38
80th	53.64	52.46	51.39	53.08	52.99	53.57	52.50	51.90	51.19	51.90	51.88	51.57	52.26
85th	54.39	53.33	52.63	54.61	54.55	54.93	54.11	53.66	53.16	53.16	53.37	53.19	53.61
90th	56.19	54.92	54.58	56.69	56.67	57.14	55.84	56.21	55.56	55.13	55.63	56.13	54.97
95th	60.00	57.20	57.14	60.31	59.49	60.58	60.13	59.24	59.74	57.96	59.09	59.08	59.68

Table S4. Waist circumference corrected for height (cm/m) percentiles in boys by age (n = 5981).

	**Age (years)**												
**Percentile**	**5 (n = 164)**	**6 (n = 442)**	**7 (n = 422)**	**8 (n = 463)**	**9 (n = 495)**	**10 (n = 502)**	**11 (n = 554)**	**12 (n = 628)**	**13 (n = 530)**	**14 (n = 521)**	**15 (n = 536)**	**16 (n = 408)**	**17 (n = 316)**
5th	42.98	41.32	40.31	40.23	39.69	40.14	39.22	37.91	38.06	37.72	37.13	37.57	39.38
10th	43.86	42.44	41.90	41.54	41.09	41.42	40.21	39.14	39.43	38.88	38.51	38.64	40.35
15th	44.44	43.70	42.62	42.22	41.91	42.03	41.33	40.25	40.31	39.77	39.41	39.43	41.14
20th	45.05	44.54	43.22	42.86	42.57	42.56	42.31	41.03	41.13	40.49	40.12	40.00	41.92
25th	45.51	45.05	43.80	43.33	43.51	43.45	43.28	41.76	41.72	40.94	40.90	40.47	42.49
30th	46.03	45.69	44.21	43.98	44.03	44.12	44.00	42.57	42.32	41.98	41.46	40.96	43.26
40th	46.79	46.61	45.38	45.16	45.56	45.38	45.39	43.86	43.67	43.04	42.50	42.42	44.58
50th	47.73	47.49	46.46	46.76	47.01	46.83	47.02	45.70	44.97	44.77	43.98	43.89	45.89
60th	48.54	48.62	48.09	48.25	48.46	48.65	49.65	47.26	46.88	46.82	45.88	45.61	47.47
70th	49.57	50.00	49.61	50.38	50.47	50.76	51.97	50.94	50.30	49.02	48.30	47.83	49.35
75th	50.81	50.91	50.42	51.39	51.94	52.48	53.21	52.27	52.04	50.92	50.00	49.28	50.61
80th	51.89	52.07	51.61	52.59	53.91	54.41	54.90	53.97	53.55	52.88	51.48	50.85	52.66
85th	53.27	53.47	53.85	54.20	55.90	56.54	56.86	56.02	55.49	54.84	53.29	52.71	54.95
90th	54.84	55.00	57.04	56.49	58.21	58.27	59.21	58.82	57.59	57.32	56.70	55.39	58.37
95th	56.78	58.87	60.00	60.00	61.43	62.06	62.40	61.99	60.61	60.24	59.66	60.24	61.20

Table S5. Waist circumference corrected for height squared (cm/m^2) percentiles in girls by age (*n* = 6998).

	Age (years)												
**Percentile**	**5 (*n* = 161)**	**6 (*n* = 477)**	**7 (*n* = 491)**	**8 (*n* = 480)**	**9 (*n* = 516)**	**10 (*n* = 549)**	**11 (*n* = 525)**	**12 (*n* = 699)**	**13 (*n* = 653)**	**14 (*n* = 666)**	**15 (*n* = 705)**	**16 (*n* = 620)**	**17 (*n* = 456)**
5th	35.67	33.71	31.00	29.74	27.63	26.23	25.27	23.92	23.87	23.98	23.03	23.44	23.05
10th	37.26	35.34	32.37	31.25	29.09	28.06	26.27	25.10	24.61	24.74	24.28	24.24	24.24
15th	38.37	36.20	33.32	32.28	29.93	28.84	26.91	25.96	25.48	25.28	25.00	24.77	24.98
20th	39.55	36.93	34.14	33.05	30.68	29.49	27.62	26.48	25.93	25.91	25.71	25.46	25.64
25th	40.30	37.50	34.91	33.52	31.36	30.08	28.21	27.02	26.60	26.53	26.35	26.11	26.37
30th	40.98	37.99	35.34	34.25	31.82	30.73	28.72	27.47	27.05	27.06	26.78	26.61	26.99
40th	42.15	39.02	36.35	35.34	32.86	31.87	29.96	28.40	27.99	28.07	27.73	27.61	27.97
50th	44.05	40.29	37.50	36.30	34.20	33.06	31.24	29.48	28.95	29.08	28.74	28.63	29.35
60th	45.44	41.59	38.78	37.59	35.50	34.24	32.42	30.63	30.04	30.00	29.71	29.76	30.49
70th	46.99	42.87	40.18	39.05	36.73	35.68	33.76	32.05	31.25	31.25	30.86	31.04	32.01
75th	47.77	43.72	40.96	39.68	37.78	36.73	34.57	32.89	32.02	32.04	31.64	32.00	32.42
80th	48.70	44.56	41.65	40.63	38.44	37.65	35.58	33.91	32.87	32.88	32.47	32.87	32.97
85th	49.43	45.44	42.66	41.57	39.66	38.46	36.45	35.20	33.88	33.78	33.75	33.88	34.21
90th	50.51	46.53	43.83	42.88	41.03	40.26	37.72	36.74	35.20	35.06	35.15	35.65	35.52
95th	53.09	48.56	45.82	44.99	43.16	43.02	40.69	38.67	38.10	36.57	37.83	38.49	37.52

Table S6. Waist circumference corrected for height squared (cm/m^2) percentiles in boys by age (n = 5981).

	Age (years)												
**Percentile**	**5 (*n* = 164)**	**6 (*n* = 442)**	**7 (*n* = 422)**	**8 (*n* = 463)**	**9 (*n* = 495)**	**10 (*n* = 502)**	**11 (*n* = 554)**	**12 (*n* = 628)**	**13 (*n* = 530)**	**14 (*n* = 521)**	**15 (*n* = 536)**	**16 (*n* = 408)**	**17 (*n* = 316)**
5th	36.35	34.20	31.36	29.90	28.85	27.55	25.84	23.77	23.22	22.13	21.31	21.37	22.09
10th	37.35	35.31	32.90	31.13	30.07	28.94	27.11	24.78	24.24	22.95	22.41	22.28	23.04
15th	38.06	36.20	33.82	32.05	30.61	29.62	28.17	25.72	24.92	23.73	22.99	22.79	23.61
20th	38.64	36.95	34.60	32.71	31.43	30.32	28.69	26.35	25.72	24.16	23.74	23.20	24.34
25th	39.94	37.50	35.20	33.48	32.05	30.75	29.42	27.19	26.15	24.86	24.11	23.58	24.76
30th	40.72	38.19	35.77	34.01	32.71	31.11	30.25	27.93	26.64	25.48	24.50	24.02	25.31
40th	41.45	39.06	36.71	35.14	33.74	32.12	31.35	29.00	27.70	26.30	25.22	24.82	25.97
50th	42.65	40.15	37.76	36.42	34.94	33.31	32.56	30.31	28.73	27.24	26.06	25.81	26.99
60th	43.61	40.98	39.00	37.59	36.13	34.85	33.79	31.44	29.96	28.58	27.31	26.71	27.97
70th	44.55	42.67	40.28	38.46	37.66	36.53	35.20	32.89	31.90	30.11	28.73	28.01	29.03
75th	45.43	43.10	40.89	39.50	38.46	37.27	36.22	33.72	32.69	30.85	29.72	28.72	30.20
80th	46.26	44.53	42.15	40.28	39.43	38.12	37.17	34.72	33.79	32.04	30.48	29.76	31.25
85th	47.85	45.42	43.07	41.54	40.70	39.54	38.37	36.33	35.06	33.31	31.75	31.07	32.05
90th	48.91	46.60	44.59	42.27	42.15	41.33	39.80	38.22	36.45	34.89	33.28	33.06	34.14
95th	50.58	48.84	47.36	44.29	44.11	43.40	41.96	40.35	38.37	37.02	35.49	35.01	35.74

Table S7. Main characteristics by sex and age group for the subsample (n = 2309).

	Age group (years)						*p*-trend §
	**5 to 6.9**	**7 to 8.9**	**9 to 10.9**	**11 to 12.9**	**13 to 14.9**	**15 to 17.9**	
	**Girls (n = 1271)**						
n	**100**	**174**	**200**	**216**	**232**	**349**	
Body weight (kg) †	21.0 a (18.6 – 24.5)	26.5 a (23.6 – 31.4)	36.5 b (29.5 – 44.5)	45.8 c (38.4 – 54.7)	53.1 d (46.8 – 63.1)	58.5 d (50.1 – 67.9)	<0.001
Height (m)	1.14 a (1.12 – 1.20)	1.26 b (1.22 – 1.31)	1.39 c (1.34 – 1.45)	1.51 d (1.46 – 1.56)	1.58 e (1.52 – 1.62)	1.60 e (1.55 – 1.64)	<0.001
BMI (kg/m^2)	15.9 a (14.7 – 17.1)	16.7 a (15.2 – 18.4)	18.6 b (16.5 – 21.9)	19.8 b (17.2 – 23.5)	21.5 c (19.1 – 25.3)	23.2 c (20.1 – 25.9)	<0.001
	**Boys (n = 1038)**						
n	**87**	**137**	**178**	**190**	**205**	**241**	
Body weight (kg)	21.6 a (19.5 – 26.6)	26.7 a (23.1 – 35.1)	36.3 b (30.5 – 44.3)	45.3 c (36.4 – 57.9)	53.4 d (46.4 – 63.8)	64.9 e (56.3 – 77.7)	<0.001
Height (m)	1.18 a (1.13 – 1.23)	1.27 a (1.23 – 1.33)	1.40 b (1.34 – 1.45)	1.50 c (1.43 – 1.57)	1.62 d (1.55 – 1.67)	1.71 e (1.66 – 1.76)	<0.001
BMI (kg/m^2)	15.8 a (14.6 – 18.6)	16.6 a (15.1 – 19.5)	18.1 b (16.4 – 21.6)	19.7 c (17.3 – 24.0)	20.3 c (18.1 – 24.3)	22.1 d (19.4 – 27.0)	<0.001

† Data expressed as median (25th – 75th percentile).
§ Calculated with Kruskal-Wallis H test.
Different letters within variables denote significant differences ($p \leq 0.05$) among age groups (Dunn's post hoc test).
BMI: Body mass index.

Table S8. Waist circumference values by sex and age group for the subsample (n = 2309).

	Age group (years)						***p*-trend §**
	**5 to 6.9**	**7 to 8.9**	**9 to 10.9**	**11 to 12.9**	**13 to 14.9**	**15 to 17.9**	
			**Girls (n = 1271)**				
n	**100**	**174**	**200**	**216**	**232**	**349**	
WC (cm) †	55.5 a<br>(52.2 – 61.0)	58.0 a<br>(54.0 – 64.3)	64.5 b<br>(58.6 – 73.0)	68.0 c<br>(62.0 – 76.0)	71.0 d<br>(65.0 – 80.0)	73.0 d<br>(67.0 – 82.0)	<0.001
WC/HT (cm/m)	48.4 a<br>(46.1 – 52.5)	46.2 b<br>(43.5 – 50.5)	46.2 b<br>(43.3 – 52.3)	44.7 b<br>(41.5 – 50.5)	45.7 b<br>(41.5 – 51.0)	46.0 b<br>(42.3 – 51.0)	<0.001
WC/HT2 (cm/m^2)	42.4 a<br>(39.5 – 46.2)	36.6 b<br>(34.6 – 40.7)	33.8 c<br>(30.9 – 37.2)	30.0 d<br>(27.6 – 33.6)	29.1 d<br>(26.5 – 32.2)	28.9 d<br>(26.4 – 32.5)	<0.001
			**Boys (n = 1038)**				
n	**87**	**137**	**178**	**190**	**205**	**241**	
WC (cm)	58.0 a<br>(54.0 – 63.5)	58.5 a<br>(54.3 – 67.0)	65.0 b<br>(58.8 – 75.0)	69.0 c<br>(62.5 – 80.0)	72.0 c,d<br>(66.5 – 83.0)	78.0 d<br>(69.0 – 88.0)	<0.001
WC/HT (cm/m)	48.0 a<br>(45.0 – 53.2)	46.9 a,b<br>(43.7 – 50.6)	46.2 a,b<br>(42.8 – 51.8)	46.1 a,b<br>(42.0 – 52.0)	44.6 b<br>(41.5 – 51.6)	45.1 b<br>(40.6 – 51.8)	<0.001
WC/HT2 (cm/m^2)	40.3 a<br>(37.8 – 46.2)	37.3 a<br>(35.0 – 40.5)	33.6 b<br>(30.7 – 37.8)	31.4 c<br>(28.4 – 35.1)	27.9 d<br>(25.5 – 32.7)	26.7 d<br>(23.7 – 30.5)	<0.001

† Data expressed as median (25th – 75th percentile).

§ Calculated with Kruskal-Wallis H test.

Different letters within variables denote significant differences between age groups (Dunn's post hoc, $p < 0.05$).

WC: Waist circumference; WC/HT: Waist circumference corrected for height; WC/HT2: Waist circumference corrected for height squared.

Table S9. Body mass index categories by sex and age group for the subsample (n = 2309).

	Age group (years)							
	**5 to 6.9**	**7 to 8.9**	**9 to 10.9**	**11 to 12.9**	**13 to 14.9**	**15 to 17.9**	***p*-trend §**	φ
	**Girls (n = 1271)**							
n	**100**	**174**	**200**	**216**	**232**	**349**		
Underweight ‡	0 (0)	3 (1.7)	3 (1.5)	3 (1.4)	6 (2.6)	10 (2.9)	0.469	0.060
Normal	76 a (76.0)	114 a,b (65.5)	108 b (54.0)	125 b (57.9)	128 b (55.2)	199 b (57.0)	0.002	0.123
Overweight	13 a (13.0)	34 a,b (19.5)	48 a,b (24.0)	52 a,b (24.1)	66 b (28.4)	99 b (28.4)	0.014	0.106
Obesity	11 (11.0)	23 (13.2)	41 (20.5)	36 (16.7)	32 (13.8)	41 (11.7)	0.073	0.089
	**Boys (n = 1038)**							
n	**87**	**137**	**178**	**190**	**205**	**241**		
Underweight	6 (6.9)	4 (2.9)	2 (1.1)	7 (3.7)	6 (2.9)	11 (4.6)	0.208	0.083
Normal	53 (60.9)	79 (57.7)	98 (55.1)	95 (50.0)	117 (57.1)	139 (57.7)	0.518	0.064
Overweight	7 a (8.0)	25 a,b (18.2)	32 a,b (18.0)	37 a,b (19.5)	55 b (26.8)	46 a,b (19.1)	0.012	0.119
Obesity	21 a,b,c (24.1)	29 a,b,c (21.2)	46 b,c (25.8)	51 c (26.8)	27 a (13.2)	45 a,b,c (18.7)	0.009	0.121

‡ Data expressed as n (%).

§ Calculated with X^2 test.

Different letters within BMI categories denote significant differences ($p \leq 0.05$) among age groups (t-test for independent proportions with Bonferroni adjustment).
